# Breast size, thoracic kyphosis & thoracic spine pain - association & relevance of bra fitting in post-menopausal women: a correlational study

**DOI:** 10.1186/2045-709X-21-20

**Published:** 2013-07-01

**Authors:** Linda Spencer, Kathy Briffa

**Affiliations:** 1 School of Physiotherapy, Faculty of Health Sciences, Curtin University, Bentley Campus, Perth, Western Australia

**Keywords:** Breast size, Thoracic spine pain, Post-menopausal women

## Abstract

**Background:**

Menopause would seem to exist as a period of accelerated changes for women and their upper torso mechanics. Whether these anthropometric changes reflect changes in pain states remains unclear. Plausible mechanisms of pain exist for the independent and combined effect of increasing breast size and thoracic kyphosis. Bra fit has the potential to change when the anthropometric measures (chest circumference and bust circumference) used to determine bra size change, such as postmenopausally.

Identifying an association between breast size, thoracic kyphosis and thoracic spine pain in postmenopausal women and identifying the relevance of bra fit to this association may be of importance to the future management and education of post-menopausal women presenting clinically with thoracic spine pain.

**Methods:**

A cross-sectional study design. Fifty-one postmenopausal bra-wearing women were recruited. Measures included breast size (Triumph International), thoracic kyphosis (flexible curve), bra fitted (Y/N) and pain (Short Form McGill Pain Questionnaire) and tenderness on palpation (posteroanterior pressure testing). These measures were collected in one session at a physiotherapy clinic.

**Results:**

The majority of the women in this study were overweight or obese and wearing an incorrect sized bra. Pain was significantly related to breast size, body weight and BMI at mid thoracic levels (T7-8). In contrast self-reported thoracic pain was not correlated with age or index of kyphosis (thoracic kyphosis). Women with thoracic pain were no more likely to have their bra professionally fitted whereas women with a higher BMI and larger breasts were more likely to have their bra professionally fitted.

**Conclusion:**

The findings of this study show that larger breasts and increased BMI are associated with thoracic pain in postmenopausal women. This is unrelated to thoracic kyphosis. Increasing breast size and how a bra is worn may have biomechanical implications for the loaded thoracic spine and surrounding musculature. Post-menopause women present with a spectrum of anthropometrical changes that have the potential to contribute to altered biomechanics and affect pain states in the thoracic spine.

## Background

Menopause represents a period of accelerated changes for women which is pivotal in the development of osteoporosis [1], obesity [2] and some pain states [3].

Ageing and menopause are closely allied but different variables. Symptoms created by both often lack definition and distinction. Changes in spinal posture and breast size are of interest in this study.

Progressive change in adult spinal sagittal posture is a well-documented characteristic feature of the ageing process [4-6]. In the thoracic spine these changes have been attributed to gradual changes in the structure and mechanics of connective tissues including loss of elasticity, a reduction in back extensor strength and concurrent collapse of vertebral bodies resulting from low bone mineral density. Accentuated kyphosis is a noticeable postural change, which has a higher incidence in women post menopause [4,6,7].

Changes in breast size have been shown to vary throughout life. Descriptive evidence suggests that breast size changes are prevalent after menopause. These changes in breast size have been described and measured in several different ways including breast mass; a breast size score; cup size and bra size. Breast mass and breast size score are indicators of absolute breast size, that is, they are independent of the size of the woman. Cup size also indicates breast size, but it is relative to the size of the woman as indicated by her under-breast chest circumference. Cup size has the advantage of being an indicator that is meaningful to most people and therefore is widely used where communication with a lay audience is important. All of these indicators are used in the literature, with the selection often influenced by the research question. For example, cup size is often used for studies of breast augmentation or reduction mammoplasty, whereas breast mass may be more appropriate for biomechanical studies. Increases in breast size post-menopause have been reported together with increases in body weight (and BMI) [8]. Mechanisms -involving a reduction in lypolitic response have been proposed for these changes [9,10]. Whilst it might be assumed that an increase in breast size might be proportionate to an increase in BMI, this has been challenged with evidence that women who lose weight after menopause still show an increase in breast size [8].

Menopause is a period of accelerated changes for women in their upper torso mechanics. Whether these anthropometrical changes reflect changes in pain states remains unclear. Plausible mechanisms for pain exist for the independent and combined effect of increasing breast size and thoracic kyphosis. Breast hypertrophy (macromastia) and associated symptomatology for example, have been well-evidenced [11]. Back pain, neck pain and discomfort during physical activity exist as predominant symptoms causing women to seek help in the form of reduction mammoplasty surgery [11]. Significant reduction of shoulder grooving, back and neck pain have been reported with a mean reduction of 2.6 cup sizes in symptomatic women post reduction mammoplasty [12]. These findings suggest that larger breast sizes do indeed equate with a greater health burden, which is amenable to change. These studies also highlight that a greater proportion of older women (menopausal age and above) undergo such surgery [11,13]. While a number of psychosocial factors might contribute to decisions by older women to have reduction surgery this might also be interpreted as a greater desire for symptomatic relief from large breasts amongst this population.

Static spinal postures have been shown to differ according to breast size [14], suggesting a biomechanical basis for some macromastic symptoms. It has been proposed that anteriorly located breast mass acts to shift the centre of gravity away from the spine thus presenting as a clinical prologue to the development of an increased cervical lordosis and thoracic kyphosis. Emotional factors might also be considered with the impact of upper torso changes as women protract the shoulders to disguise cup size. The addition of a poorly fitting bra with tight shoulder straps and excessive elevation of breast tissue acts to increase the downward drag of breast weight and alter mechanics further [15]. Pain produced by such mechanisms would intuitively be dependent on the size and weight of the breast and surrounding cutaneous tissue, which potentially implicates overall body weight & BMI in the symptom pathway.

A large proportion of large breasted women wear incorrectly sized bras [16]. To date bra fit appears unrelated to pain in normal healthy individuals despite the theory that a poorly fitting bra would compromise bra function (breast support and minimisation of breast bounce) and contribute to unfavourable spinal mechanics [16]. Pain related to bra fit may manifest as shoulder grooving, back or neck pain and neuropathic symptoms in the upper limbs.

Eliciting whether bra fit contributes to pain in an ‘at risk’ population may be more fruitful. Bra fit has the potential to change when the anthropometrical measures (chest circumference and bust circumference) used to determine bra size change, such as post-menopausally. One in five women have reported that after menopause they had to buy a larger bra because of changes in breast size [8]. Evidence has identified that women show a concomitant increase in thoracic kyphosis and BMI post-menopausally which may also alter chest circumference measures. Therefore, both breast size and chest circumference are likely to change post-menopausally. This may result in the redundancy of a previously well-fitting bra and an appearance of macromastic symptomatology which operates through the mechanism of an ill-fitting bra. It however, remains unknown whether a woman changes cup size, band size or both when selecting a new bra post-menopusally.

In this study we examined thoracic spine pain and tenderness in relation to breast size, thoracic kyphosis and bra fit in post-menopausal women in order to begin exploring the question of whether breast size, thoracic kyphosis and bra fit have consistent independent or combined associations with thoracic spine pain in this population. Clarification of these associations may aid the care of postmenopausal women presenting with thoracic pain.

## Methods

This study was approved by the Human Research Ethics Committee at Curtin University. All participants provided written informed consent for their participation in the study.

### Participants

Fifty-one post-menopausal women (50-84 years) with and without thoracic pain, who wore a bra on a daily basis, volunteered to participate in this study. Post-menopause was defined as having had their last menstrual period more than one year ago [17]. Thoracic pain was defined as pain felt anywhere in the posterior aspect of the thoracic cage (region bordered by the first thoracic vertebrae and rib superiorly, and the twelfth thoracic vertebrae and rib inferiorly).

Recruitment posters for this study, displayed in community halls and physiotherapy clinics in the Mandurah local area, invited post-menopausal women, who wore a bra on a daily basis to volunteer for this study. Volunteers were excluded if they: (a) were not post-menopausal, (b) had undergone any form of thoracic spine surgery, (c) had a known pathology involving the breast, lung or thoracic spine, (d) had had any form of cancer, (d) were using or had had prolonged use of steroid medication, or (e) were unable to read or understand English.

### Data collection and measures

Data collection for this study comprised two parts:

(1) Participants completed a screening questionnaire to ensure inclusion criteria were met and to collect descriptive data, bra size and fitting details. This self-report measure identified the current bra size worn by the women and whether they routinely had their bra fitted by a trained professional (yes/no response).

(2) Eligible participants attended a Physiotherapy clinic on one occasion for the collection of anthropometrical (breast size & thoracic kyphosis) and thoracic pain measurements.

#### Breast size

Correct bra size was established for each woman according to Triumph guidelines using under-bust and over-bust circumferences [18]. Under-bust circumference (band size) indicated the bra size (10 to 20, analogous to sizes for other clothing) and the difference between under- and over-bust circumferences indicated cup-size (A to G). A tabulated conversion chart provided by Triumph Australia was used to convert the measured bra size to a continuous breast size score (3 to 15). These scores are indicative of cup volume, and consequently breast volume, independent of the band size of the woman and based on calculations of the cup/breast volume as the volume of half a sphere [19]. This method is analogous to those used in the sizing of breast prostheses whereby a size of 4 (for example) is approximately equivalent to a breast volume of 400 cm^3^. Tabulated conversions simplify application of the system [18] (see Additional file 1 for calculation table adapted from Triumph). It appears this method was used in a previous study examining relationships between breast size, bra fit and thoracic pain [16]. The application of a continuous numerical scale in less sophisticated ways to indicate cup size is also apparent in literature examining breast size [20]. The bra size measured according to the Triumph guidelines was compared with the self-reported bra size to indicate whether or not the participants in this study were wearing a bra of the correct size.

#### Thoracic kyphosis

A Flexible Curve (Faber-Castell, Germany) was used to measure the size of the sagittal plane curve (i.e. kyphosis) in the thoracic spine. Denoted by an index of Kyphosis (IK), with higher scores indicating greater degrees of thoracic kyphosis. The intra- and inter-rater reliability of flexible curve measures of thoracic kyphosis are acceptable with reported coefficients of 0.88 and higher [21,22].

#### Thoracic spine pain

*Short form McGill Pain Questionnaire (SF-MPQ)*[23]*:* A total pain score (0-38) was derived from the three subscales (Sensory, Affective & Visual Analogue Scale) of the short form McGill Pain Questionnaire. Satisfactory test–retest reliability and responsiveness values of the NSF-MPQ have been reported [24].

#### Tenderness on palpation

*PA Pressure Testing:* Provocation and the behaviour of localised tenderness in the thoracic spine was assessed using a central posteroanterior (PA) applied pressure (grade III) through the spinous processes of the thoracic (T4-10) spine [25]. Each vertebrae producing tenderness with a large- amplitude, through-range oscillatory mobilisation after ten seconds or less were recorded.

### Data analysis

Data were analysed using SPSS version 17. Univariate and multivariate relationships between breast size, thoracic kyphosis (IK) and thoracic spine pain (SF-MPQ) were examined using Pearson’s correlation co-efficients (r) and stepwise multiple regression analysis respectively.

Tenderness at each thoracic spinal segment tested was plotted using frequency histograms. T-tests were used to compare breast size variables between women with and without tenderness at each thoracic vertebra.

Chi squared analyses were used to examine associations between the categorical variables of bra fitted and thoracic tenderness on palpation at each level. The relationship between breast size and the likelihood of having bras fitted was examined with logistic regression analysis.

Level of significance was set as p < 0.05. There were no adjustments for multiple comparisons as these were likely to increase the chances of type II error [26].

## Results

Fifty-one women (50-84 years) volunteered for this study.

Seven women were excluded. Six because they did not meet the selection criteria and one because she did not return the screening questionnaire. Consequently data from 44 women were included in the analyses.

**Figure 1:**
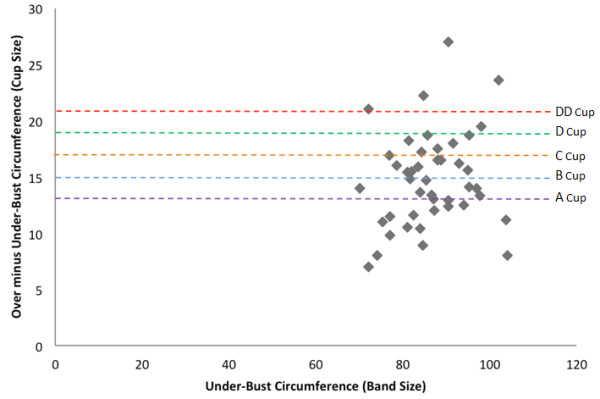
Distribution of bra sizes.

The majority of the sample were overweight (32%) (BMI 25-30) or obese (23%) (BMI >30) (Table 1). No women were underweight. Bra sizes ranged from 10A to 22E (median 16B). The average under-bust circumference (band size) was 87 cm and over-bust circumference 102, equivalent to a bra size of 16C (Figure 1). Thoracic Kyphosis indices ranged from 5.9 to 19.1 with a mean thoracic kyphosis of 11.7. Thoracic pain scores ranged from 0 to 38 with a mean score of 8.3 on the SF-MPQ.

**Table 1:** Descriptive data

	**Mean (SD)**
Age	69 (9.1)
Height	162 (5.0)
Weight	71 (11.0)
BMI	26.5 (4.7)
Breast size score	7.8 (2.8)
Pain(SF-MPQ)	8.3 (8.7)
Thoracic kyphosis(IK)	11.7 (2.9)

Note: *IK* Index of Kyphosis.

*SK*-MPQ Short form Mc Gill pain questionnaire.

Mean (standard deviation) shown.

Just over half of the women sampled (52%) reported having their bra fitted. The expense associated with bra fitting and the perception that women were able to self-select bras reasonably well were the main reasons given by participants in this study for not having their bra fitted.

Comparisons between measured bra size and bra size worn regardless of whether or not the bras worn had been professionally fitted suggested that the majority (93%) of participants were wearing incorrectly sized bras, with 57% wearing bras that were too small and 36% wearing bras that were too big. The brand of bra worn and the timing of when participants last had their bra fitted were not known.

**Figure 2:**
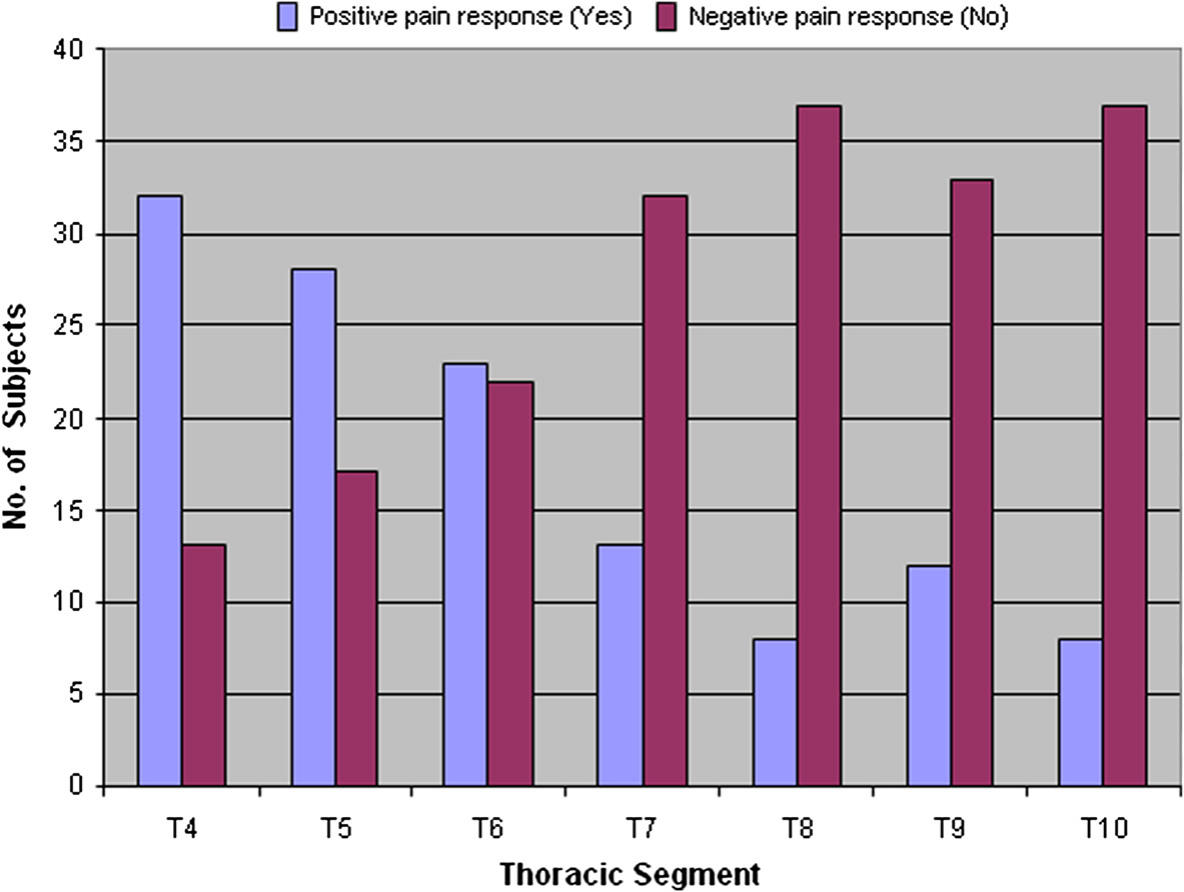
Thoracic spine segmental pain frequency.

Tenderness on palpation was more prevalent at higher thoracic levels with 73% reporting pain at T4, decreasing to 18% reporting pain at T10 (Figure 2). There was no difference in the index of kyphosis or BMI between those with or without tenderness on palpation at any of the thoracic levels (p > 0.05). Breast size was significantly associated with pain at T7 (p = 0.007) and T8 (p = 0.02).

There was a positive correlation between body weight and breast size (Table 2). This was mirrored by a strong positive correlation between BMI and breast size. Body weight, BMI and breast size were correlated with thoracic pain indicating that larger women with larger breasts reported more thoracic pain. In contrast, thoracic pain was not correlated with age or thoracic kyphosis (IK) (Table 2).

**Table 2:** Relationships (Pearson’s) between age, anthropometric variables and self reported thoracic pain

	**Weight**	**BMI**	**IK**	**Breastr size**	**Pain**
Age	−0.26 (p = 0.09)	−0.22 (p = 0.16)	−1.0 (p = 0.34)	−0.11 (p = 0.48)	−0.14 (p = 0.35)
Weight		0.88 (p < 0.001)	0.066 (p = 0.68)	0.67 (p < 0.001)	0.47 (p = 0.001)
BMI			0.03 (p = 0.87)	0.57 (p < 0.001)	0.41 (p = 0.005)
IK				0.20 (p = 0.20)	0.08 (p = 0.60)
Breast size					0.46 (p = 0.002)

Note: *IK* Index of Kyphosis.

P = values are from Pearson’s correlation co-efficients calculated between variables.

In stepwise multiple regression analysis including breast size, IK, and BMI as independent variables, only breast size was significantly associated with thoracic pain (p = 0.002).

Women with thoracic spine pain were no more or less likely to have their bra fitted (p = 0.31) than women without pain. In contrast, women with larger breasts were more likely to have their bra fitted. For every 1-point increase in breast size score, a woman was 1.3 times more likely to get her bra fitted (CI = 1.04-1.74).

## Discussion

The purpose of this study was to explore the relationship between breast size, thoracic kyphosis, bra fit and thoracic pain in post-menopausal women. Menopause and ageing are similar but distinct variables. Menopause exists as period of rapid transition for women in terms of lean tissue mass [27], bone mineral density [28], fat tissue distribution [27], and a range of subsequent anthropometrical dimensions. Identifying if and how these changes might impact on pain experienced by post-menopausal women will help direct future clinical practice.

The sample of women who participated in this study were relatively healthy, community dwelling individuals who were not self-selected on the basis of seeking treatment for pain.

The majority of women were overweight (BMI > 25-30) or obese (BMI > 30), which is coherent with national statistics showing this to be the largest and most rapidly expanding category in women, particularly post-menopausally [29]. Body weight and BMI were significantly associated with breast size, indicating somewhat intuitively that larger women have larger breasts. Women with a greater BMI and larger breasts were more likely to have their bra fitted but the majority of women in this study were not wearing the correct bra size.

Wearing incorrectly sized bras has also been reported in young women with thoracic pain [16]. The procedure for fitting a bra is reasonably universal but due to the constraints on how it can be measured, some random error cannot be excluded in the process. Assumed differences between bra brands together with other substantiated variables such as measurement methodology and chest expansion during measuring [30] are factors affecting the inter-rater reliability in bra measurements.

Breast hypertrophy has been associated with menopause with many women reporting an increase in breast size post-menopause [8]. Women of post-menopausal age are more likely to seek reduction mammoplasty surgery [11,13], suggesting that the postmenopausal increase in breast size is pertinent to symptoms leading women to seek such surgery. There was large variation in the bra size of participants in this study, varying as it did from 10A to 22E with an average size of 16C. Whilst there is no consensus on the definition of macromastia, women with bra cup sizes exceeding DD appear most frequently in reduction mammoplasty studies [11]. If bra cup size is taken as an indicator of breast size, it might be assumed that the DD cup size is a threshold at, or above which, common symptoms associated with macromastia develop. This might be usefully regarded by clinicians treating this category of women but further clarification is needed as to whether breast size, mass, shape and position are influential in part or combination to this relationship.

Large breast size has been purported to exist as a significant health burden in some postmenopausal women, operating mainly through the mechanism of pain [13]. The two procedures used for the assessment of pain in this study provided both localised and global subjective indications of thoracic tenderness and pain respectively. A significant moderate relationship was found between breast size and global thoracic pain. Localised tenderness on palpation at the thoracic levels T7 and T8 was significantly associated with breast size. Although a series of t-tests was used to examine these relationships and no corrections for multiple comparisons were implemented, the p-value for T7 was very small (p < 0.007) and the finding of significant associations at two adjacent levels add plausibility to the finding.

Whilst a causal relationship between breast size and thoracic pain (particularly at T7 and T8) cannot be concluded from these cross-sectional data, it is encouraging to find that existing literature has highlighted potential mechanisms that are consistent with the findings of this study. The pulley-like action of brassiere straps and associated downward drag of breast weight has been implicated in the cause of pain in the scapular elevator muscles [15]. It might be speculated that scapular retractor muscles (regional to T7/8) are also a potential source of pain as the shoulder girdle protracts with a larger anterior load (larger breasts). These findings and those produced in the present study provide anatomical, clinical and statistical confirmation and consensus of the plausibility of such a mechanism and highlight the mid thoracic region as one which is implicated with increased stress afforded by larger breasts. Further investigation into the shape and volume of breast tissue in post-menopausal women would help clarify the specific biomechanical impact of breast size relative to overall upper body size/fat distribution which could be used to further elucidate possible pain mechanisms.

Collectively the results of this study support the general consensus of reduction mammoplasty research indicating that breast size is indeed instrumental in producing symptoms of upper back pain in post-menopausal women. With the majority of women undergoing breast reduction surgery being overweight or obese [13] and knowing that pain prevalence has been linked to obesity [31] with the relative odds of chronic pain increasing with every unit of BMI [32], it is helpful that the present study has identified an association between breast size and pain which appears independent of body weight and BMI. If breast size had not been shown to be the most important independent predictor of thoracic pain in this study then it might be speculated that the improvements in symptoms seen in reduction mammoplasty studies are less contingent on actual breast size but more so on breast weight, particularly since some studies report the removal of up to 2330 g per breast [12]. This could arguably translate into a somewhat significant immediate reduction in body weight (and BMI) post-surgery and thus explain the symptom improvement based on the aforementioned relationships.

The results of this study indicate that upper thoracic pain exists in women of postmenopausal age with 82% of participants indicating some pain in the upper back (SF-MPQ score >0). Progressive change in adult spinal sagittal posture is a characteristic feature of the ageing process which is accentuated in women, around the menopause through osteoporosis [6]. Conversely, It is now well-established that the sagittal curvatures of the spine are geometric parameters that are known to have a significant influence on mechanical properties during compression loading [33,34]. Sagittal posture imbalance has been shown to elevate loads and intervertebral disc stresses, thus posing as a plausible mechanism for pain in persons with an increased thoracic kyphosis [6]. Whilst the results of this study cannot confirm the association between increasing kyphosis and global or localised thoracic pain in post-menopausal women, it does highlight the existence of thoracic pain in this population. Breast size is associated with this pain at thoracic levels T7 and T8, which are levels that have been previously identified in postmenopausal women as being frequently deformed (due to wedging or fracture) [4,6] and close to the apex of kyphosis (T6) [5]. Given the abundance of existing literature that has identified relationships between increasing age [6], BMI [7] and pain [35] with accentuated thoracic kyphosis, further examination of these variables in post-menopausal women is suggested.

In summary, the findings of this study show a correlation between breast size and upper back pain in postmenopausal women. This is unrelated to thoracic kyphosis and appears independent of co-existing associations between increasing BMI and thoracic pain. Whilst women with larger breasts are more likely to have their bra fitted, this appears unrelated to thoracic pain.

This study has identified a handful of correlates associated with thoracic pain in post-menopausal women. It is acknowledged that additional possible correlates exist and in this study it was not possible to account for all of these. Bone mineral density, physical activity levels, emotion and in some instances, occupational stresses, are factors that were not measured and may have confounded our results. Random error associated with breast size and thoracic kyphosis measurement should be considered as limitations of this study. In addition, the results of this study were reliant on the accuracy of responses to self-reported measures of pain, bra wear and fitting. Collectively the under or over-reporting of details pertaining to these measures cannot be discounted and should also be considered limitations of this study.

Further research is required to examine women postmenopausally. It exists as a transitional time for women in terms of breast size and BMI, which could also reflect a transitional time in terms of pain. An intuitive and proven relationship exists between breast size and body weight and this is related to thoracic pain. Elucidating possible mechanisms to explain these relationships would be a logical progression to understanding why they exist and how clinical practice might be improved in the treatment of post-menopausal women presenting with thoracic pain.

## Conclusions

Multiple factors could determine the prevalence of thoracic pain in postmenopausal women. Based on the results of this study, breast size, body weight, BMI appear important. Thoracic kyphosis and whether the bra is of correct size show little meaningful correlation with thoracic pain in this population. Additional potentially causal factors are plentiful and inter-related. Whilst many of these factors are a feature of normal ageing, most are amenable to change and potentially responsive to conservative measures and therefore are worthy of future investigation.

## Competing interests

The authors declare that they have no competing interests.

## Authors’ contributions

This study was completed by LS in partial fulfilment of a Masters degree. KB acted as supervisor to this study. LS conceived the idea for the study. LS and KB designed the study and sought ethical approval. LS collected the data. LS and KB analysed the data. All authors contributed to the writing of this manuscript. All authors read and approved the final manuscript.

## Supplementary Material

Additional file 1 **The calculation table shown below was used in the conversion of bra size to a breast size score (1-15) **[18] This elicited an estimate of breast volume and provided a continuous variable for statistical analysis. Click here for file
