# Supplementary figures and images for: Breast size, thoracic kyphosis & thoracic spine pain - association & relevance of bra fitting in post-menopausal women: a correlational study
Source: Chiropr Man Therap. 2013 Jul 1;21:20. doi: 10.1186/2045-709X-21-20 (PMC3704920; doi:10.1186/2045-709X-21-20)

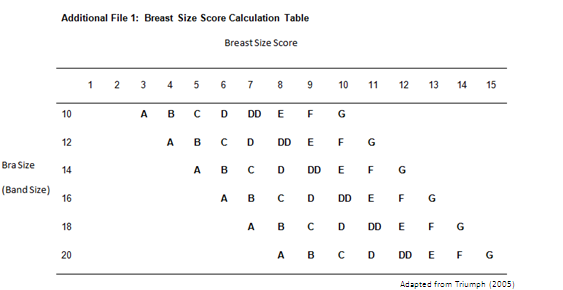

Supplement: Additional file 1 — The calculation table shown below was used in the conversion of bra size to a breast size score (1-15) [18] This elicited an estimate of breast volume and provided a continuous variable for statistical analysis. [file 2045-709X-21-20-S1.bmp]
